# Topics of the Month

**Published:** 1893-05

**Authors:** 


### BUFFALO MEDICAL AND SURGICAL JOURNAL

A MONTHLY REVIEW OF MEDICINE AND SURGERY.

*EDITORS:*

THOMAS LOTHROP, M. D. - - WM. WARREN POTTER, M. D.

All communications, whether of a literary or business character, should be addressed to the managing editor: 284 FRANKLIN STREET, BUFFALO, N. Y.

VOL. XXXII. MAY, 1893. No. 10.

### TOPICS OF THE MONTH.

A BILL introduced in the last Legislature, but which was, unfortunately, buried in committee, known as the Southworth bill, created no little anxiety and worry among patent medicine manufacturers and their greatest friends,—not the people, but the newspapers. The bill conferred upon the State Board of Health the power to analyze and examine all drugs and medicines known as patent or proprietary medicines, and the conditions and regulations under which such drugs and medicines should be sold to the public. As was to be expected, the newspapers, with their usual eye to business, made the greatest remonstrance against this measure, some even going so far as to insinuate that the bill was instigated by jealous practitioners. No one for a moment doubts that if the provisions of this bill could be carried out, the cork and bottom of the patent medicine business would fall out—both at the same time—and "suffering humanity" would be thereby increased, while the public would be proportionately benefited and protected. Medicines whose formulæ will not bear publication will not bear honest trial, and hence they are only intended to deceive and delude mankind at a time when he, above all times, should have honest advice and scientific treatment by a conscientious and intelligent physician. Exactly similar laws are in vogue in countries less highly civilized than our own, and what is good law for them might be good law for us.

———

THE interesting paper from the pen of Mr. Marcy, on The Responsibility of the Physician to the Patient, Considered from a Legal Point of View, is worthy of most careful attention. Physicians are, at times, careless in the attention and treatment of their charity cases, and imagine that, because they are not certain of a compensation, their part of the contract can be neglected. It is but recently that a suit of damages was instituted, and carried through successfully, against a prominent Pittsburg physician, who was, perhaps, somewhat careless in his attention to a charity patient.

The following extract from Mr. Marcy's paper should be well borne in mind by physicians who so generously devote their time and skill for the alleviation of the suffering poor:

It is also to be observed that the same rules and principles apply when the service is gratuitous, and the physician who has undertaken a case may not leave his patient without giving full and ample opportunity to call in some other when he retires.

---

Too much credit cannot be given our efficient Health Officer, Dr. Wende, for the vigilance and scrutiny which he has shown in protecting the public health interests of Buffalo. His determined fight and ultimate success in every cause that has attracted his attention is remarkable when we consider his opponents in the city's council chambers. The passage of the Tenement-house ordinances is the latest and, perhaps, the greatest victory. In this reform he was ably assisted by Dr. Pryor. Now that they are passed, no one dare doubt of their rigid enforcement.

The Journal would suggest to Dr. Wende the advisability of publishing in its columns a monthly report of the mortality and natal statistics of the city, such as is carried out in Cincinnati and other cities. The Journal would cheerfully publish such reports, and hopes it may soon be able to do so.

---

A movement is on foot for the erection of an international monument to the memory of Professor J. Ph. Semmelweis, who died in Vienna in 1865. The Medical Society of Budapest has taken the matter in hand, and has appointed an international committee to solicit funds for this purpose. It is proposed to erect the monument in Budapest. The treasurer of the committee is Dr. Elischer, Place petöfi, Budapest, Hungary.

---

The attending and consulting staffs of the Buffalo City Hospital have resigned as a body. The reason assigned for this action is, that the doctors tried to make it a *private* institution for the public gain, whereas the management persisted in making it a *public* institution for *private* gain.

THE common enemy must be getting very formidable and dangerous when the Erie County Medical Society and the Erie County Homeopathic Society meet on common ground to discuss ways and means for his subjugation. It shows, nevertheless, a healthy and a more liberal state of feeling than has existed in bygone days. The meeting held at the Young Men's Christian Association building, Thursday evening, April 20th, was a successful and a timely one, but whether it carried much weight with the political powers that be, is a question which time alone can tell.

THE March-April number of the Saint Joseph, Mo., *Medical Herald* is, in every respect, a very handsome and interesting production. It was published as a souvenir edition, complimentary to the Medical Society of the Missouri Valley, and if the society is not sufficiently appreciative of this worthy token of respect, then it certainly ought to disband.

